# Comprehensive Analysis of Liver Transcriptome and Metabolome Response to Oncogenic Marek’s Disease Virus Infection in Wenchang Chickens

**DOI:** 10.3390/biology14080938

**Published:** 2025-07-25

**Authors:** Lifeng Zhi, Xiangdong Xu, Yang Zeng, Wenquan Qin, Ganghua Li, Junming Zhao, Runfeng Zhang, Guang Rong

**Affiliations:** 1 Hubei Key Laboratory of Edible Wild Plants Conservation and Utilization, College of Life Sciences, Hubei Normal University, Huangshi 435002, China; 2 Tropical Crops Genetic Resources Institute, Chinese Academy of Tropical Agricultural Sciences, Danzhou 571700, China; 3 Key Lab of Tropical Crops Information Technology Application Research of Hainan Province, Institute of Science and Technology Information, Chinese Academy of Tropical Agricultural Sciences, Haikou 571101, China

**Keywords:** Marek’s disease virus, host response, transcriptomics, metabolomics, chicken

## Abstract

Marek’s disease (MD), induced by the highly contagious Marek’s disease virus (MDV), remains a significant challenge to global poultry health despite extensive vaccination efforts. This study employed integrated transcriptomic and metabolomic analyses to investigate liver responses in naturally MDV-infected Wenchang chickens during late infection stages. RNA sequencing identified 959 differentially expressed genes (DEGs) between the infected and uninfected groups. Functional enrichment analysis demonstrated that these DEGs were primarily associated with canonical pathways related to metabolism and cellular processes, including lipid, carbohydrate, and amino acid metabolism, as well as the p53 signaling pathway, cell cycle, and apoptosis. Ultra-high-performance liquid chromatography coupled with quadrupole time-of-flight mass spectrometry (UPLC-Q-TOF-MS) detected 561 differentially expressed metabolites (DEMs), showing near-significant enrichment (*p* = 0.069) in phenylalanine metabolism. Integrated analysis of transcriptomics and metabolomics data highlighted that critical gene–metabolite pairs such as SGPL1-palmitaldehyde–sphinganine-1-phosphate and ME1-NADP+–malic acid potentially mediate functional crosstalk between sphingolipid metabolism and cellular redox homeostasis during viral oncogenesis. This comprehensive mapping of regulatory networks provides insights into host–virus interactions during MDV pathogenesis, offering potential applications in immunomodulation approaches, targeted therapeutic strategies, and vaccine adjuvant development.

## Simple Summary

Marek’s disease is a serious illness in chickens caused by a highly contagious virus, continuing to threaten poultry farms worldwide despite existing vaccines. To understand how the virus damages chickens’ livers in later stages of infection, we studied naturally infected Wenchang chickens using advanced molecular analysis tools. We discovered 959 differentially expressed genes and 561 dysregulated metabolites in infected birds. These changes reveal how the virus subverts hepatic energy metabolism—disrupting how they process fats, sugars, and proteins—and interferes with vital cell functions like growth control and programmed cell death. Most notably, we identified specific enzyme–metabolite interactions (like the SGPL1 enzyme with sphingolipids) that help the virus weaken cells’ defenses while promoting tumor growth. By mapping these functional correlations between genes and metabolites, our study uncovers how the virus modulates host molecular pathways. These findings could lead to new strategies for disease control, such as improved treatments, precision vaccines, and potentially designing disease-resistant chickens, ultimately protecting farmers’ livelihoods and safeguarding poultry production.

## 1. Introduction

Marek’s disease (MD) is a highly contagious avian disorder caused by the oncogenic alphaherpesvirus Marek’s disease virus (MDV; *Gallid alphaherpesvirus* 2). Despite decades of vaccination, it remains a global threat to poultry health. MDV induces fatal T-cell lymphomas, immunosuppression, and neurological disorders, with annual economic losses exceeding USD 1 billion due to mortality and reduced productivity in vaccinated flocks [1]. MDV is classified into three serotypes (1, 2, and 3), with serotype 1 strains being exclusively oncogenic. Worryingly, the increasing virulence of MDV has triggered outbreaks even in vaccinated flocks, challenging current vaccine strategies and resulting in major economic losses. MDV infection initiates through the inhalation of virus-laden feather dust and dander, followed by productive-lytic replication in B lymphocytes and macrophages and latent infection of activated T lymphocytes, predominantly CD4+ T cells, facilitated by the viral oncogene *Meq* (methylene blue-stainable protein) and host immune evasion mechanisms such as MHC-I downregulation [2]. Subsequently, MDV can induce neoplastic transformation of latently infected T-cells, leading to the development of T-cell lymphomas, the hallmark of classical Marek’s disease. The viral infection cycle and its pathogenic consequences involve complex interactions between viral gene expression and host metabolic reprogramming.

Considerable efforts have been made in recent years to investigate host responses to MDV infection, focusing on selective breeds including the experimental White Leghorn (layer) lines 6_3_ and 7_2_, which are MD-resistant and susceptible, respectively. Genome-wide quantitative trait loci (QTLs) with MD resistance or susceptibility were mapped on chromosomes 1, 5, 7, 9, 15, 18, 26, Z, E21, and E16 [3,4,5]. More than 5000 genes were dysregulated in the liver, thymus, spleen, or bursa of Fabricius of MDV-infected chickens at different stages by RNA sequencing technology coupled with quantitative real-time PCR [6,7,8,9]. Chicken genetic resistance/susceptibility to MDV is strongly associated with major histocompatibility complex (MHC)—B haplotypes, interferon-γ (IFN-γ) polymorphisms, and dysregulated cytokine networks (e.g., IL-8 and IL-18) [10]. Key functions, such as antigen presentation, interferon (IFN) response, and cell apoptosis, and key pathways, including JAK-STAT signaling and Toll-like receptor (TLR)-mediated innate immunity, are critical for viral containment during early infection [11]. However, immunosuppressive viral proteins (e.g., vIL-8) and metabolic hijacking of glutathione and lipid biosynthesis pathways compromise host defenses during late infection [12,13,14,15].

Existing studies predominantly utilize controlled laboratory infections with standardized chicken lines (e.g., SPF White Leghorns), which inadequately reflect field conditions where genetic diversity, environmental stressors, and co-infections modulate disease outcomes [4]. Over-reliance on artificial infection models (e.g., intra-abdominal inoculation) fails to recapitulate natural transmission dynamics, while a focus on early infection stages overlooks critical tumorigenic phases. The immunometabolic landscape of MDV-infected chickens under commercial farming conditions remains poorly characterized, particularly during late-stage tumorigenesis. Although the liver plays a key role in antiviral metabolism, its involvement in field-relevant host responses—such as detoxification and redox regulation—is seldom studied. This study employs integrated transcriptomics and metabolomics to profile hepatic responses in naturally MDV-infected Wenchang chickens—a local breed with uncharacterized MDV susceptibility—during late infection stages. By correlating dysregulated genes with altered metabolic pathways, we aim to identify immunometabolic checkpoints governing tumor progression in field settings.

## 2. Materials and Methods

### 2.1. Animal Husbandry and Sample Collection

The Wenchang broilers were hatched in a single batch and raised in two separate houses under uniform conditions at the farming base of Yexiang Wenchang Chicken Breeding Co., Ltd. in Wenchang, Hainan, China. By 60 days of age, 30% of the birds in one house exhibited classic clinical signs such as lethargy, glazed eyes, paralysis, anorexia, and severe weight loss. At 70 days, five sick birds with at least two clinical signs in the sick house and five healthy chickens in the other house without clinical signs were selected. They were humanely euthanized via CO_2_ asphyxiation (30% chamber volume/min) followed by confirmatory cervical dislocation. Post mortem pathological examination revealed that the livers of the affected chickens were enlarged and featured grayish-white tumor lesions on their surfaces, whereas no significant lesions were observed in the livers of the asymptomatic chickens. Liver tissue samples were collected and immediately frozen in liquid nitrogen for subsequent analysis. Concurrently, anticoagulant blood samples were collected to test for Avian leukosis virus (ALV).

### 2.2. Transcriptomic Analysis

After exclusion of ALV infection by using ELISA (GB/T 26436-2010, the Chinese ALV disease diagnostic methods, Beijing, 2010 [16]) and conformation of MDV infection using PCR methods (GB/T18643-2021, the Chinese Marek’s disease diagnostic methods, Beijing, 2021 [17]), total RNA was extracted from liver tissue using the TRIzol reagent (Invitrogen, Carlsbad, CA, USA) according to the manufacturer’s protocol. RNA integrity and concentration were assessed using a NanoDrop One spectrophotometer (Thermo Fisher Scientific, Waltham, MA, USA) and an Agilent 2100 Bioanalyzer (Agilent Technologies, Santa Clara, CA, USA). High-quality RNA samples with an RNA integrity number (RIN) value > 7.5 were then used to construct sequencing libraries using the NEBNext Ultra Directional RNA Library Prep Kit (New England Biolabs, Ipswich, MA, USA). Sequencing was performed on an Illumina HiSeq2000 platform (Illumina, San Diego, CA, USA).

After removing adapter sequences, duplicated reads, and low-quality reads, the clean reads were aligned to the *Gallus gallus* reference genome GRCg6a (GCA_000002315.5) using HISAT2 (v2.0.5) with default parameters [18]. The uniquely mapped reads were quantified using HTSeq (2.0.2) [19]. Differentially expressed genes (DEGs) were identified using DESeq2 [20], with a threshold of |log2FoldChange| ≥ 1 and a *p*-value ≤ 0.05. DEGs were subjected to Gene Ontology (GO) term and KEGG pathway enrichment analyses using topGO [21] and clusterProfiler [22], respectively. The DEG interaction network was constructed using NetworkAnalyst [23], based on protein–protein interactions obtained from STRING (Search Tool for the Retrieval of Interacting Genes/Proteins, version 11.5).

### 2.3. Quantitative Real-Time PCR (qRT-PCR)

Ten candidate genes were selected for validation of the RNA-seq results using quantitative real-time PCR (qRT-PCR) (Table S1). Complementary DNA (cDNA) was synthesized from total RNA using the HiScript^®^ Q RT SuperMix for qPCR (+gDNA wiper) (Vazyme, Nanjing, China) according to the manufacturer’s instructions. qRT-PCR amplifications were performed in triplicate using a QuantStudio™ 5 Real-Time PCR System (Applied Biosystems, Foster City, CA, USA). Each 10 μL reaction mixture contained 2.0 μL cDNA (100 ng), 0.25 μL of each primer (forward and reverse, 10 nM), 5.0 μL of 2 × ChamQ SYBR qPCR Master Mix (Vazyme, Nanjing, China), and 2.5 μL of nuclease-free water. The cycling conditions were as follows: an initial denaturation at 95 °C for 30 s, followed by 40 cycles of denaturation at 95 °C for 10 s, annealing at 60 °C for 30 s, and extension at 72 °C for 20 s. Relative gene expression was calculated using the 2^−ΔΔCt^ method, with glyceraldehyde-3-phosphate dehydrogenase (GAPDH) serving as a reference gene.

### 2.4. Metabolomic Analysis

Liver samples were defrosted at 4 °C. Flesh samples were homogenized with a grinding pestle. An amount of 100 μL of the homogenized sample was mixed with 1000 μL of methanol. The mixture was then homogenized for one minute. The samples were then centrifuged at 12,000 rpm for 10 min at 4 °C. The supernatant was transferred, and the supernatant was evaporated to dryness. The residue was reconstituted with 200 μL of a 2-chloro-L-phenylalanine solution (4 ppm) prepared in 80% methanol–water. The extract was then filtered through a 0.22 μm filter and transferred to a vial for subsequent liquid chromatography–tandem mass spectrometry (LC-MS) analysis.

Metabolite profiling of the liver tissue samples was conducted by PANOMIX (Suzhou, China) using an LC-MS system comprising a Vanquish UHPLC System coupled to an Orbitrap Exploris 120 mass spectrometer (Thermo Fisher Scientific, USA). Chromatographic separation was achieved on an ACQUITY UPLC^®^ HSS T3 column (2.1 × 100 mm, 1.8 μm, Waters, Milford, MA, USA), maintained at 40 °C. The autosampler was set to a temperature of 4 °C, with a flow rate of 0.3 mL/min and an injection volume of 2 μL [24]. Mass spectrometric detection of metabolites was performed on an Orbitrap Exploris 120 (Thermo Fisher Scientific, USA) with an electrospray ionization (ESI) source [25]. Full MS-ddMS2 acquisition was used to acquire simultaneous MS1 and MS/MS spectra. Metabolite identification was performed based on accurate mass measurements and MS/MS fragmentation patterns, with cross-referencing against the Human Metabolome Database (HMDB), MassBank, LipidMaps, and mzCloud, as well as the PANOMIX (Shuzhou, China) metabolite database. Unsupervised (PCA) and supervised (PLS-DA and OPLS-DA) multivariate statistical analyses were applied using the Ropls (v1.22.0) package to discriminate between groups [26]. Significantly altered metabolites (DEMs) were selected using VIP > 1 and a *p*-value < 0.05.

Metabolic pathway analysis with the hypergeometric distribution enrichment method was performed using KEGG (http://www.genome.jp/kegg/, accessed on 10 March 2025) and MetaboAnalyst (http://www.metaboanalyst.ca/, accessed on 15 March 2025) [27].

### 2.5. Transcriptome and Metabolome Association Analysis

Pearson correlation coefficients were calculated to assess the relationship between DEGs and DEMs. All DEGs and metabolites were mapped to the KEGG pathway database to identify common pathway information. The main biochemical and signal transduction pathways were then analyzed. Gene and metabolite networks were constructed and visualized using igraph software (Version 1.6.0) [28].

## 3. Results

### 3.1. Transcriptomic Alterations Induced by MDV Infection

**Figure 1:**
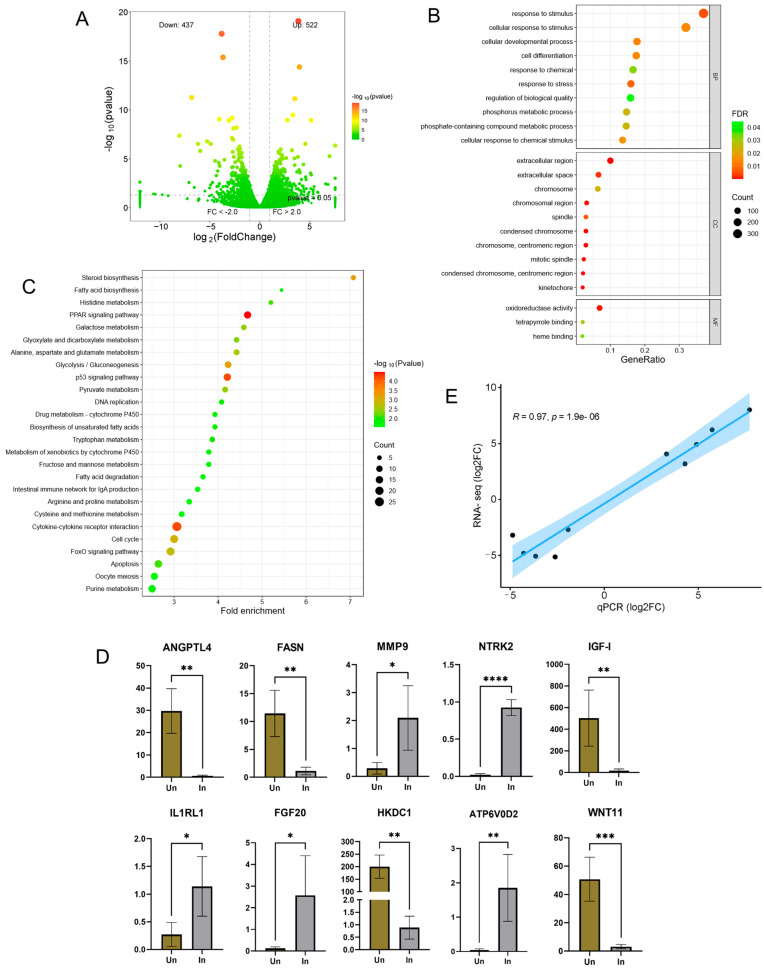
Transcriptomic alterations to MDV infection in livers of Wenchang chickens: (**A**) volcano plot; (**B**) GO enrichment of differentially expressed genes; (**C**) KEGG pathway enrichment of differentially expressed genes; (**D**) validation of differential gene expression via qPCR (mean ± SD, *n* = 5 biological replicates). Statistical significance was determined by Student’s *t*-test (*p* < 0.05). Sample labels: Un = uninfected controls, In = MDV-infected cohort. Significance denoted: * *p* < 0.05; ** *p* < 0.01; *** *p* < 0.001; **** *p* < 0.0001. (**E**) Correlation of RNA-seq results and qPCR results.

In this study, a total of 69.22 Gb of clean data was obtained after quality control from 71.07 Gb of Illumina sequencing data. Approximately 95.58% of clean reads (±2.71%, SD) was mapped to the chicken genome assembly using HISAT2. A total of 959 genes were differentially expressed between the infected and uninfected groups, including 522 upregulated and 437 downregulated genes (Figure 1A, Table S2).

The gene enrichment analysis identified a total of 205 GO terms in response to the infection (FDR < 0.05), including 186 biological process (BPs), 15 cellular components (CCs), and 3 molecular function terms (Figure 1B, Table S3). Many important GO terms are mainly involved in cell growth and death and metabolism and immunity. The upregulated and downregulated genes were enriched separately to identify certain expression patterns (Figure S1, Tables S4 and S5). The upregulated genes were found to be significantly enriched in cell growth and death and immunity-related terms, such as cellular developmental process (GO:0048869), cell differentiation (GO:0030154), cell cycle (GO:0007049), response to stress (GO:0006950), immune system process (GO:0002376), and immune response (GO:0006955). The downregulated genes were significantly classified into metabolism-related terms, such as lipid metabolic process (GO:0006629), carbohydrate metabolic process (GO:0005975), fatty acid metabolic process (GO:0006631), and nucleoside bisphosphate metabolic process (GO:0033865). Only one GO term, extracellular region (GO:0005576), was significantly enriched by both the upregulated and downregulated genes.

A total of 26 KEGG pathways were significantly impacted by MDV infection between the infected and uninfected groups (Figure 1C, Table S6). These pathways primarily related to various biomolecules of metabolism, including carbohydrate, lipid, amino acid and nucleotide, and cell growth and death of cellular processes, such as p53 signaling pathway (gga04115), cell cycle (gga04110), apoptosis (gga04210), and oocyte meiosis (gga04114). The upregulated gene enrichment analysis identified several significant canonical pathways (Figure S2A, Table S7). Among these, four pathways, p53 signaling pathway (gga04115), cell cycle (gga04110), apoptosis (gga04210), and oocyte meiosis (gga04114), were categorized into cell growth and death of cellular processes, in good agreement with all the DEG enrichment results. Three pathways, intestinal immune network for IgA production (gga04672), NOD-like receptor signaling pathway (gga04621), and cytosolic DNA-sensing pathway (gga04623), may play important roles in the immune systems of the infected birds. Of note, downregulated genes provided distinct enrichment patterns (Figure S2B, Table S8). Seventeen of twenty-two significantly enriched pathways were identified to function in biomolecule metabolism, and three pathways may function in signal transduction. Only one pathway, the PPAR signaling pathway (gga03320), was significantly impacted by both the upregulated and downregulated genes.

The expression results of the RNA-seq analysis were validated by qRT-PCR in the same sample set (Figure 1D). The correlation coefficient (R) of the log2 transformed fold change values between the qRT-PCR and RNA-seq platforms was 0.97 (*p* = 1.9 × 10^−6^) (Figure 1E). The expression results of ANGPTL4, ATP6V0D2, FASN, FGF20, HKDC1, IGF-I, IL1RL1, MMP9, NTRK2, and WNT11 obtained using qRT-PCR were in good agreement with the RNA-seq results (*p*-value < 0.001).

### 3.2. Alterations in Metabolism Induced by MDV Infection

**Figure 2:**
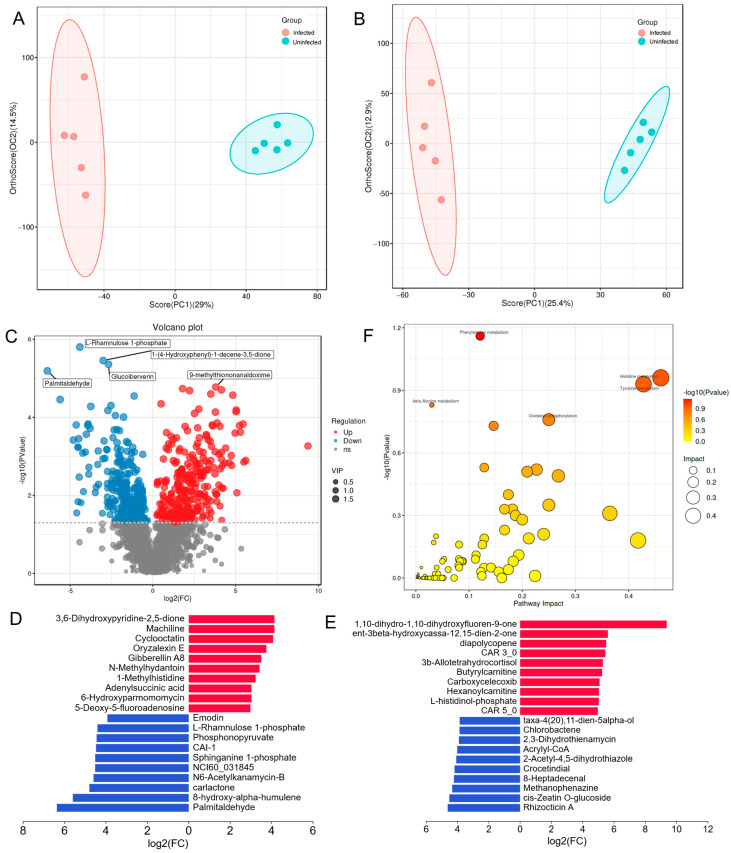
Metabolomic alterations to MDV infection in livers of Wenchang chickens: (**A**,**B**) OPLS-DA plot in the positive model and the negative model, respectively; (**C**) volcano plot of differentially expressed metabolites; (**D**,**E**) the top 20 metabolites in the positive model and the negative model, respectively; (**F**) KEGG annotation of differentially expressed metabolites.

Untargeted metabolomics was performed to understand the alterations in the metabolites of livers between the infected and uninfected chickens. Multivariate statistical analysis revealed a clear separation of metabolites between the infected and uninfected groups in the negative model (Figure 2A) and the positive model (Figure 2B). Cross-validation demonstrated robust model performance across ionization modes (negative: R^2^Y = 0.994/Q^2^ = 0.77; positive: R^2^Y = 0.99/Q^2^ = 0.816), where Q^2^ > 0.5 confirms the predictive capability and R^2^Y − Q^2^ < 0.3 validates model stability without overfitting. A total of 561 DEMs (375 in the negative model and 186 in the positive model) were identified by setting thresholds VIP > 1 and *p*-value < 0.05, including 288 upregulated and 273 downregulated DEMs (Figure 2C, Table S9). Among these DEMs, 1,10-dihydro-1,10-dihydroxyfluoren-9-one, ent-3beta-hydroxycassa-12,15-dien-2-one, rhizocticin A, and cis-zeatin O-glucoside in the positive model and 3,6-dihydroxypyridine-2,5-dione, machiline, palmitaldehyde, and 8-hydroxy-alpha-humulene in the negative model were the top contributors to the group separation (Figure 2D,E). All DEMs were subjected to KEGG annotation analysis and, only phenylalanine metabolism (gga00360) was nearly significantly enriched (*p* = 0.069) (Figure 2F, Table S10).

### 3.3. Comprehensive Analysis of the Transcriptomics and Metabolomics

**Figure 3:**
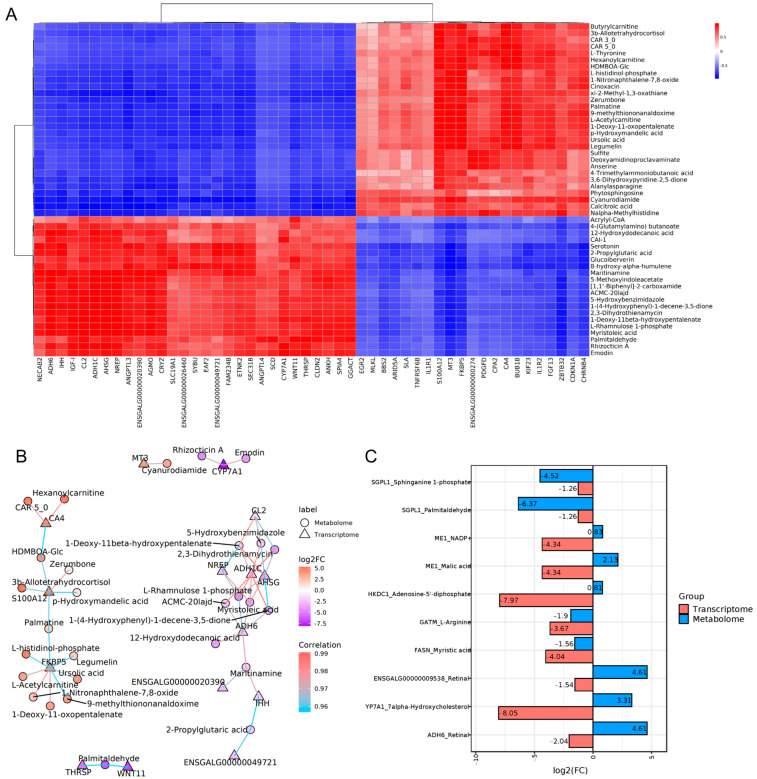
Comprehensive analysis of the transcriptomics and metabolomics in livers of Wenchang chickens: (**A**) heatmap of the correlation between significantly expressed genes and significantly expressed metabolites (top 50); (**B**) network diagram of DEGs and DEMs; (**C**) diagram of the corresponding relationship between enzymes and metabolites.

Based on Pearman calculations, the correlation of the DEGs from transcriptomics and the DEMs from metabolomics were measured. Correlations > 0.8 and a *p*-value < 0.05 were considered as significantly correlated gene–metabolite pairs, and the correlation heatmap and network of top 50 differential metabolites and differential genes were shown in Figure 3A,B (Table S11). No common KEGG pathway was found to be significantly enriched with either the DEGs or DEMs. The DEGs and DEMs were mapped to the KEGG enzyme database to explore the correspondence between metabolites in metabolic processes and the expression regulation of enzymes, and the top 10 corresponding relationships were displayed in Figure 3C based on the magnitude of the |log2(FC)| values (Table S12). SGPL1 pairing with palmitaldehyde and sphinganine 1-phosphate and ME1 pairing with NADP+ and malic acid were the most representative among these corresponding relationships.

## 4. Discussion

This study investigated host immune responses during tumorigenesis following Marek’s disease virus (MDV) natural infection through an integrated analysis of transcriptomic and metabolomic profiles in hepatic tissues from the Chinese indigenous Wenchang chicken breed. Our multi-omics approach revealed that MDV infection profoundly modulated the expression landscape of hepatic genes and metabolites, particularly influencing metabolic- and immune-related pathways. The coordinated downregulation of metabolic pathways coupled with the upregulation of immune-related networks suggests that the host appears to suppress metabolism while activating immune responses, likely redirecting energy toward antiviral defense and limiting virus-induced tumor development. Notably, we identified critical gene–metabolite pairs through correlation network analysis, including SGPL1-palmitaldehyde–sphinganine-1-phosphate and ME1-NADP+–malic acid axes, which may mediate functional crosstalk between sphingolipid metabolism and cellular redox homeostasis and likely contribute to the immunometabolic adaptation during viral oncogenesis, as evidenced by their significant enrichment in cytokine signaling pathways and T-cell activation processes. The comprehensive mapping of these regulatory networks provides mechanistic insights into host–virus interactions during MDV pathogenesis, offering potential applications in immunomodulation approaches, targeted therapeutic strategies, and vaccine adjuvant development.

Close examinations of the transcriptomic changes in response to MDV infection revealed that hepatic tissue in the host exhibited a significant downregulation of numerous genes. This suppression primarily occurred through impaired pathways associated with lipid, carbohydrate, and amino acid metabolism, collectively leading to systemic metabolic inhibition. Concurrently, upregulated genes demonstrated prominent functional enrichment in biological processes related to cell growth and death and immune system. These activated pathways facilitated enhanced immune surveillance and response mechanisms, thereby enabling the host to more effectively combat MDV infection and mitigate tumor progression. Tumor expansion and progression may induce the deprivation of oxygen and nutrients. The resulting metabolic stress causes tumor necrosis, which leads to oxidative-stress-induced cell death [29]. Among these differentially expressed genes, twelve genes significantly influenced by infection were associated with tumor necrosis factor production, comprising eight upregulated and four downregulated in the infected group when compared to the uninfected group (Table S13). Notably, GPNMB, a constitutively expressed type 1 transmembrane glycoprotein found across most cell types and tissues and involved in the maturation of hematopoietic and lymphoid cells and the activation of T lymphocytes [30], exhibited a substantial upregulation (188.57-fold) in the infected birds. Additionally, the mRNA expression of SPON2, a member of the extracellular matrix protein family used as a broad-spectrum tumor marker and implicated in the regulation of tumor metastasis and progression [31,32], was significantly altered by infection (24.99-fold). Conversely, the transcript levels of IGF-I, EPHB2, and CD36 were 33.33-, 2.70-, and 2.63-fold higher in uninfected animals than infected animals (Table S14). Of particular interest, 66 genes associated with oxidoreductase activity were significantly affected by infection. The expression of these oxidoreductase-activity-related genes was predominantly reduced in infected birds relative to their uninfected counterparts (Table S15). Among these, 48 genes were significantly downregulated in infected birds, including CYP7A1 (265.50-fold), SCD (62.08-fold), ME1 (20.31-fold), FASN (16.44-fold), and CYP2C18L (16.27-fold), while 18 genes were upregulated, such as MICAL2, CYP1C1, AKR1B10, RRM2, and IL4I1. The concerted suppression of oxidoreductases establishes a pro-oxidant milieu that potentiates MDV-induced genomic instability and immune evasion—mechanistically akin to redox dysregulation in HTLV-1 and EBV oncogenesis. Targeting this axis may disrupt MDV’s latency-to-tumor transition, though functional validation remains necessary.

Mapping of differential genes and metabolites to the KEGG enzyme database may provide insight into host–virus interactions during MDV infection and tumor progression. The association between sphingosine-1-phosphate lyase 1 (SGPL1) and the metabolites palmitic aldehyde/sphinganine 1-phosphate ranked among the top 10 enzyme–substrate relationships in hepatic metabolism. In mammalian systems, ceramide (Cer) undergoes decarboxylation catalyzed by ceramide synthase to generate sphingosine (Sph), which is subsequently phosphorylated by sphingosine kinases (SphKs) to form the bioactive lipid sphingosine-1-phosphate (S1P). SGPL1 then irreversibly cleaves S1P into phosphoethanolamine [33]. S1P exerts dual regulatory functions through both extracellular and intracellular mechanisms. Extracellularly, it binds to S1P receptors (S1PRs) via autocrine/paracrine signaling, activating downstream pathways including Ras/ERK1/2 and PI3K/Akt to mediate cellular proliferation, migration, inflammatory cytokine infiltration, angiogenesis, and autoimmune responses [34]. Intracellularly, S1P acts as a second messenger, directly binding to targets of histone deacetylases (HDACs), TNF receptor-associated factor 2 (TRAF2), to modulate calcium homeostasis, gene transcription, and protein modification. Emerging evidence reveals S1P’s critical role in tumor progression through autophagy regulation. During early tumorigenesis, S1P-mediated autophagy demonstrates tumor-suppressive effects. In lymphangioleiomyomatosis, activated SphK1/S1P/S1PR3 signaling promotes mTORC1 activation and autophagy suppression, reducing tumor cell survival, migration, and invasion [34]. In multiple myeloma, S1P deficiency reduces cell survival via autophagy [35]. Conversely, in advanced malignancies, S1P induces cytoprotective autophagy that facilitates tumor progression. In prostate cancer cells, extracellular S1P activates S1PR5, which triggers autophagy through endoplasmic reticulum stress [36,37]. Breast cancer MCF-7 cells exhibit SphK1-dependent elevation of intracellular S1P that promotes pro-survival autophagy [38,39]. Notably, SGPL1-mediated S1P degradation in hepatocellular and colorectal carcinomas disrupts S1P signaling, suppressing autophagy and accelerating carcinogenesis [40]. Our study reveals the significant downregulation of both S1P and SGPL1 in Wenchang chicken livers during late-stage MDV infection. This suggests a potential host strategy to inhibit tumorigenesis through the suppression of S1P-mediated pro-tumorigenic autophagy, though further mechanistic validation is required. These findings align with previous reports of S1P’s dichotomous roles in cancer biology and emphasize the therapeutic potential of targeting the S1P–autophagy axis in viral oncogenesis.

The enzymatic interaction between ME1 and its cognate substrates NADP+/malic acid emerged as a top decile-ranked pairing in splenic metabolic networks, exhibiting distinct spatial colocalization patterns compared to SGPL1-mediated pathways. Malic enzyme (ME) converts malate to pyruvate while producing NADPH, playing a key role in malate metabolism [41]. ME1, the main cytosolic form, links glycolysis to the TCA cycle via pyruvate production and supports fatty acid synthesis and glutamine metabolism by supplying NADPH [42]. Elevated ME1 expression has been documented across multiple malignancies [43]. As a master regulator of NADPH homeostasis, ME1 fuels tumor cell survival under metabolic stress by sustaining glutamine recycling, fatty acid biosynthesis, glycolytic flux, and reactive oxygen species (ROS) detoxification [44]. Mechanistically, ME1-derived NADPH coordinates cancer cell proliferation, invasion, apoptosis resistance, and metabolic adaptation through redox balance maintenance and anabolic pathway activation [45]. Our data reveal a marked upregulation of NADP+ and malic acid coupled with significant ME1 suppression in Wenchang chicken livers during late-stage MDV infection. This inverse correlation suggests a potential host-driven anti-tumor mechanism involving ME1 inhibition to disrupt NADPH-dependent pro-tumorigenic pathways. Given ME1’s established role in sustaining cancer cell survival under energy stress [42], its downregulation may impair tumor cell redox homeostasis and biosynthetic capacity, thereby limiting oncogenic progression. Further functional studies are warranted to validate this hypothesis and elucidate the precise molecular mechanisms.

## 5. Conclusions

Based on integrated transcriptomic and metabolomic analyses, this study delineates hepatic responses in naturally infected Wenchang chickens during late-stage infection. We identified 959 differentially expressed genes and 561 dysregulated metabolites, revealing profound disruption in lipid/carbohydrate/amino acid metabolism, p53 signaling, and apoptosis. Critically, multi-omics integration exposed novel gene–metabolite regulatory axes—SGPL1-palmitaldehyde–sphinganine-1-phosphate and ME1-NADP+–malic acid—that coordinate sphingolipid remodeling and redox imbalance during viral oncogenesis. These molecular hubs functionally bridge immunosuppression with tumorigenesis, explaining MDV’s ability to subvert host defenses despite immune priming. By mapping these mechanisms in a natural infection model, our work provides evidence that metabolic rewiring sustains MDV persistence beyond infection. These insights establish a framework for targeting host-directed pathways (e.g., sphingolipid inhibition) to develop adjuvant therapies that complement existing vaccines, offering new strategies to reduce economic losses in poultry production systems worldwide.

## Data Availability

The raw RNA-seq data are available in the National Genomics Data Center (NGDC) Genome Sequence Archive (GSA: PRJCA040775) (https://ngdc.cncb.ac.cn/gsa, accessed on 30 December 2024).

## Supplementary Materials

The following supporting information can be downloaded at https://www.mdpi.com/article/10.3390/biology14080938/s1, Table S1: Primers for qPCR; Table S2: DEGs; Tables S3–S5: GO enrichment for DEGs; Tables S6–S8: KEGG enrichment for DEGs; Tables S9 and S10: DEMs and KEGG enrichment for DEMs; Tables S11 and S12: Top 50 DEM and DEG correlation/top 10 corresponding relationships of DEMs and DEGs; Tables S13–S15: DEGs enriched in tumor necrosis factor production, oxidoreductase activity, and extracellular region; Figure S1: GO enrichment of up-DEGs and down-DEGs; Figure S2: KEGG pathway enrichment of up-DEGs and down-DEGs.
