# Supplementary figures and images for: Comprehensive Analysis of Liver Transcriptome and Metabolome Response to Oncogenic Marek’s Disease Virus Infection in Wenchang Chickens
Source: Biology (Basel). 2025 Jul 25;14(8):938. doi: 10.3390/biology14080938 (PMC12383417; doi:10.3390/biology14080938)

## Slide 1
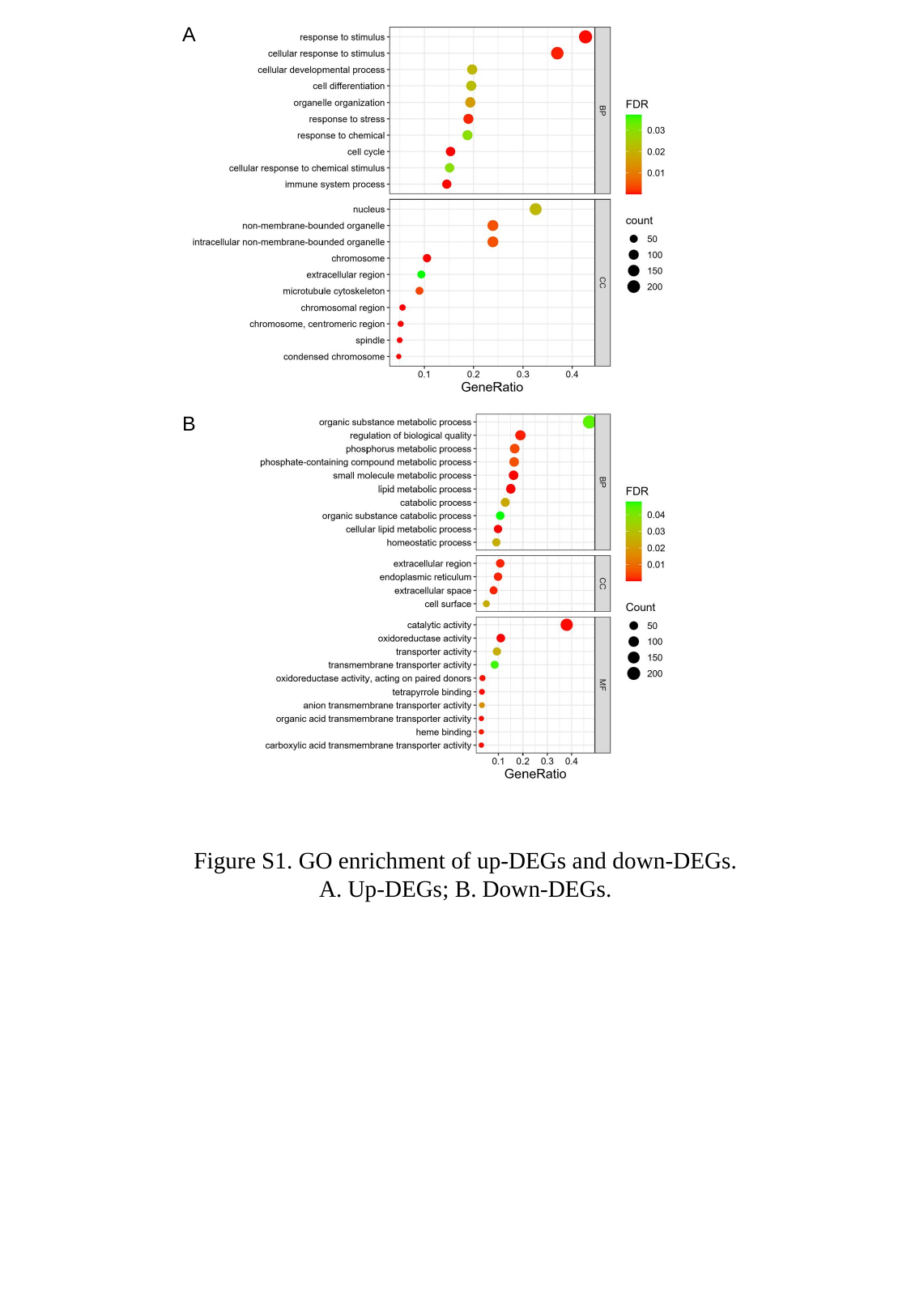

A
B
Figure S1. GO enrichment of up-DEGs and down-DEGs.
A. Up-DEGs; B. Down-DEGs.

Supplement: Supplementary file 1 [file biology-14-00938-s001.zip › Figure S1 GO enrichment of up-DEGs and down-DEGs.pptx]

## Slide 1
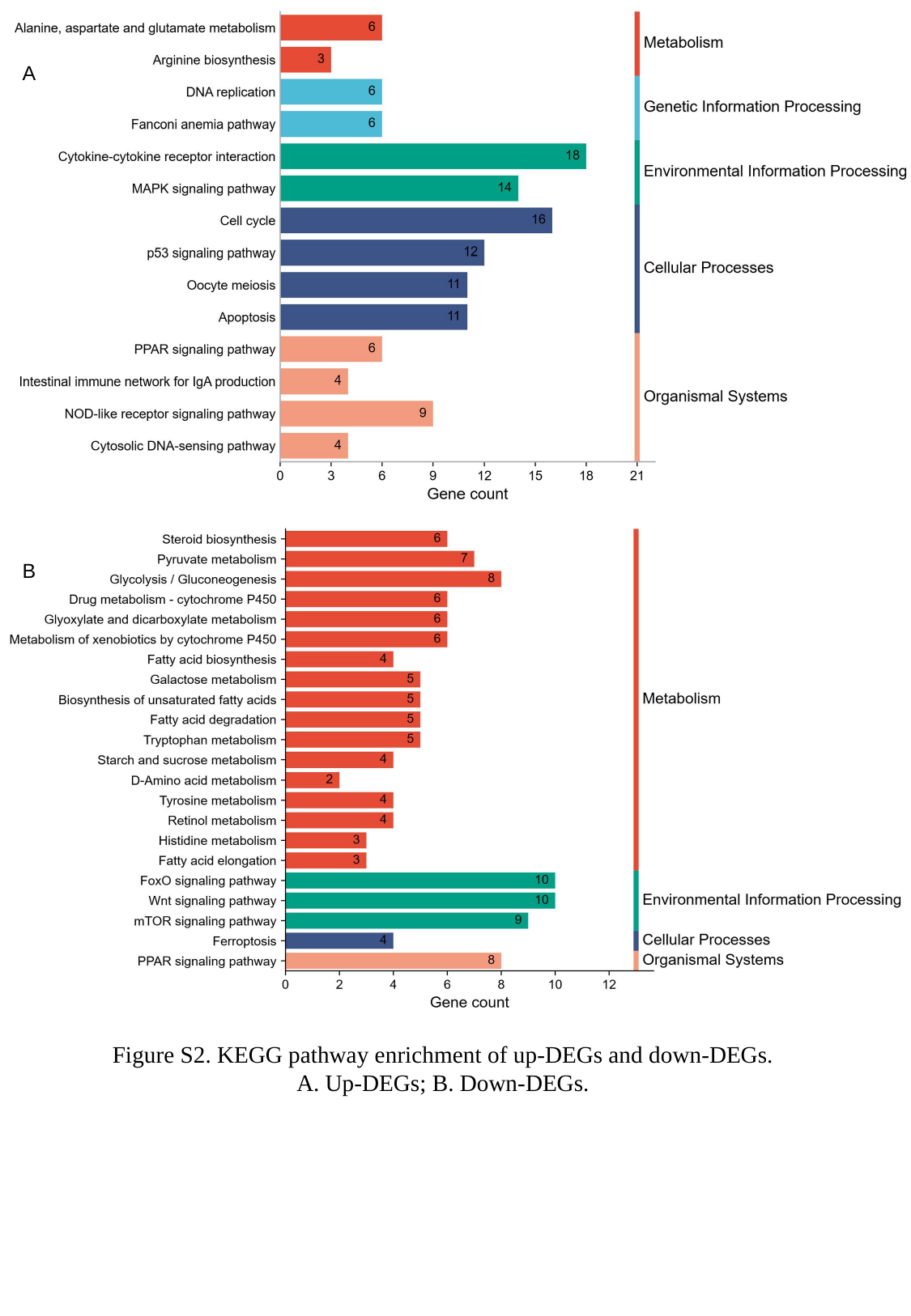

A
B
Figure S2. KEGG pathway enrichment of up-DEGs and down-DEGs.
A. Up-DEGs; B. Down-DEGs.

Supplement: Supplementary file 1 [file biology-14-00938-s001.zip › Figure S2 KEGG pathway enrichment of up-DEGs and down-DEGs.pptx]
